# CRF receptor 1 antagonism and brain distribution of active components contribute to the ameliorative effect of rikkunshito on stress-induced anorexia

**DOI:** 10.1038/srep27516

**Published:** 2016-06-08

**Authors:** Sachiko Mogami, Chiharu Sadakane, Miwa Nahata, Yasuharu Mizuhara, Chihiro Yamada, Tomohisa Hattori, Hiroshi Takeda

**Affiliations:** 1 Tsumura Research Laboratories, Tsumura & Co., Ibaraki 300-1192, Japan; 2 Pathophysiology and Therapeutics, Faculty of Pharmaceutical Sciences, Hokkaido University, Sapporo, Hokkaido 060-0812, Japan; 3 Hokkaido University Hospital Gastroenterological Medicine, Sapporo, Hokkaido 060-8648, Japan

## Abstract

Rikkunshito (RKT), a Kampo medicine, has been reported to show an ameliorative effect on sustained hypophagia after novelty stress exposure in aged mice through serotonin 2C receptor (5-HT_2C_R) antagonism. We aimed to determine (1) whether the activation of anorexigenic neurons, corticotropin-releasing factor (CRF), and pro-opiomelanocortin (POMC) neurons, is involved in the initiation of hypophagia induced by novelty stress in aged mice; (2) whether the ameliorative effect of RKT is associated with CRF and POMC neurons and downstream signal transduction; and (3) the plasma and brain distribution of the active components of RKT. The administration of RKT or 5-HT_2C_R, CRF receptor 1 (CRFR1), and melanocortin-4 receptor antagonists significantly restored the decreased food intake observed in aged male C57BL/6 mice in the early stage after novelty stress exposure. Seven components of RKT exhibited antagonistic activity against CRFR1. Hesperetin and isoliquiritigenin, which showed antagonistic effects against both CRFR1 and 5-HT_2C_R, were distributed in the plasma and brain of male Sprague-Dawley rats after a single oral administration of RKT. In conclusion, the ameliorative effect of RKT in this model is assumed to be at least partly due to brain-distributed active components possessing 5-HT_2C_R and CRFR1 antagonistic activities.

Late-life anxiety and depression is a social problem in aging societies, and the recognition of this condition and adequate treatment are increasingly required^1^. Adverse life events are associated with the onset and persistence of depression^2^, which is characterized by feeding abnormalities (anorexia and bulimia). In particular, the co-occurrence of depression and anorexia may influence morbidity and progressive physical disability in the elderly^3^,^4^.

Japanese traditional (herbal) or “Kampo” medicines are standardized with regard to the quality and quantity of their ingredients and have been approved by the Japanese Ministry of Health and Welfare. At present, almost 90% of physicians in Japan use Kampo medicines in their daily practice, sometimes as the first choice for treatment^5^,^6^. Rikkunshito (RKT) is a Kampo medicine that is often prescribed for anorexia and upper gastrointestinal disorders^7^,^8^,^9^. In addition, several multicenter, double-blind, randomized placebo-controlled studies have been conducted to examine its effects^10^,^11^. Various non-clinical studies of RKT have also been performed and have demonstrated its ameliorative effects on hypophagia and gastrointestinal dysmotility^12^. We have previously shown that RKT improves the sustained decrease in food intake that occurs after novelty stress exposure in aged mice and that its effect are associated with serotonin 2C receptor (5-HT_2C_R) antagonism^13^,^14^. It has also been reported that several RKT components possess 5-HT_2C_R antagonistic activities *in vitro*^13^,^15^. However, the inhibitory activities of these components are assumed to be insufficient to explain the complete set of effects of RKT, indicating the involvement of another mechanism of action.

There is intimate interplay between 5-HT_2C_Rs and anorexigenic corticotropin-releasing factor (CRF) neurons. The administration of 5-HT_2C_R agonists leads to increased levels of stress hormones in the blood^16^. Moreover, CRF-containing neurons in the paraventricular nucleus of the hypothalamus (PVN) co-express 5-HT_2C_R mRNA^17^. Conversely, the CRF-induced decrease in orexigenic hormone secretion is restored by 5-HT_2C_R antagonism^18^, indicating that CRF and 5-HT_2C_R signals influence each other. Furthermore, it has been reported that 5-HT_2C_R is also expressed in anorexigenic pro-opiomelanocortin (POMC) neurons in the arcuate nucleus and that 5-HT_2C_R activation promotes α-melanocyte-stimulating hormone (α-MSH) production^19^,^20^,^21^. This production contributes to appetite suppression via melanocortin-4 receptor (MC4R) activation. However, it remains to be determined whether the ameliorative effects of RKT are associated with these anorexigenic neurons and downstream signal transduction. Furthermore, whether RKT components exert any effects on CRF receptors (CRFRs) or MC4R is unclear to date.

Various active components of RKT have been previously identified, and their 5-HT_2C_R antagonistic effects have been described^13^,^15^. Recently, the plasma levels of 32 RKT components were characterized in healthy volunteers after a single oral administration^22^. However, it is crucial to determine whether the active components of RKT penetrate the blood–brain barrier (BBB) if RKT exerts its effect on CRF and POMC neurons in the brain that are considered to be involved in the stress response. The brain distribution of RKT components in humans can only be estimated based on their distribution in the cerebrospinal fluid or determined through positron emission tomography. However, such analyses are difficult to perform for RKT because it contains various types of components.

The present study was conducted to determine 1) whether the activation of anorexigenic CRF or POMC neurons is involved in the initiation of hypophagia induced by novelty stress in aged mice; 2) whether the ameliorative effect of RKT is associated with anorexigenic CRF or POMC neurons and downstream signal transduction; and 3) the plasma and brain distributions of RKT active components.

## Results

### Effects of RKT and a 5-HT_2C_R antagonist on the decrease in cumulative food intake in aged male mice after novelty stress exposure under fasting conditions

**Figure 1:**
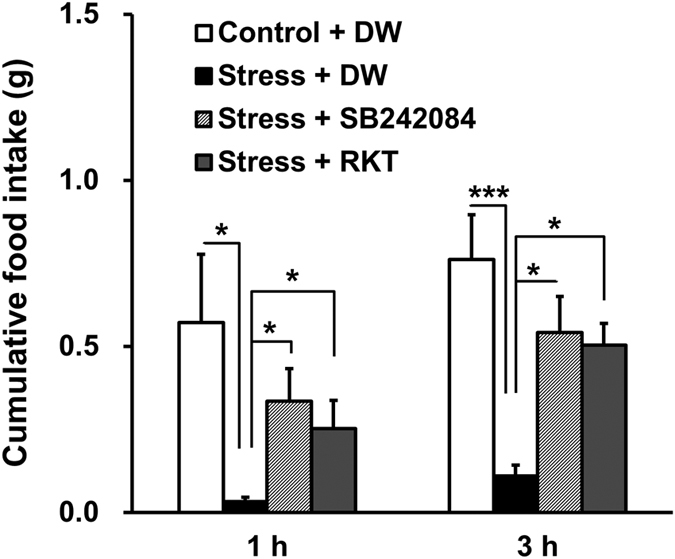
Effect of RKT and a 5-HT_2C_R antagonist on novelty stress-induced hypophagia. Aged male mice (77 ± 8 weeks old) were deprived of food overnight and orally administered the 5-HT_2C_R antagonist SB242084 (6 mg kg^−1^), RKT (1000 mg kg^−1^) or DW. Immediately after administration, the mice were exposed to novelty stress and their cumulative food intake was measured. The data are presented as the mean ± SEM (*n* = 5). *^,^***p < 0.05, 0.001, respectively, vs. the stress group based on Steel’s test at 1 h and Dunnett’s test at 3 h. DW, distilled water; RKT, rikkunshito.

Cumulative food intake over 1 and 3 h was significantly decreased by novelty stress exposure in aged male mice (p = 0.025 and 0.0004, respectively), and this decrease was significantly restored by the oral administration of RKT (p = 0.025 and 0.023, respectively) or the 5-HT_2C_R antagonist SB242084 (p = 0.025 and 0.013, respectively) at 1 and 3 h after novelty stress exposure (Fig. 1).

### Effects of CRFR1/2 or MC4R antagonists on the decrease in cumulative food intake in aged male mice after novelty stress exposure under fasting conditions

**Figure 2:**
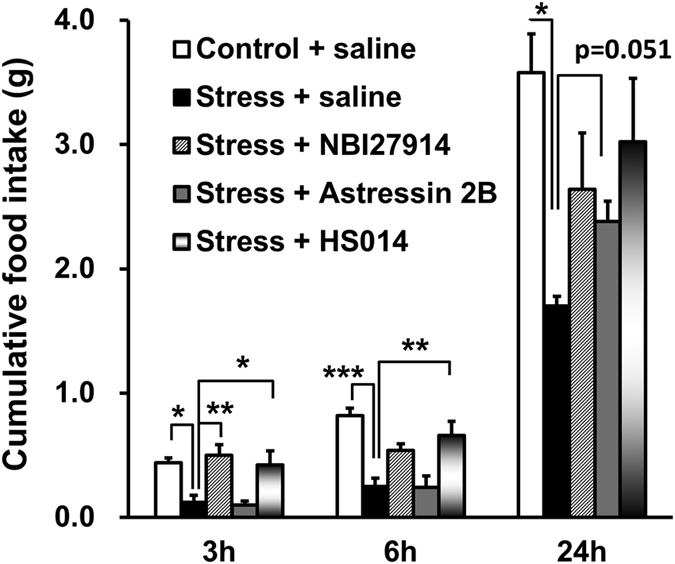
Effects of intracerebroventricular (ICV) administration of CRFR antagonists and an MC4R antagonist on novelty stress-induced hypophagia. Aged male mice (77 ± 8 weeks old) were deprived of food overnight, and a CRFR1 antagonist (NBI27914, 10 μg per mouse), a CRFR2 antagonist (astressin 2B, 10 μg per mouse), an MC4R antagonist (HS014, 0.15 μg per mouse) or sterilized physiological saline was administered via ICV injection. Immediately after administration, the mice were exposed to novelty stress and their cumulative food intake was measured. The data are presented as the mean ± SEM (*n* = 5, except for the stress group at 6 h; *n* = 4). *^,^**^,^***p < 0.05, 0.01, 0.001, respectively, vs. the stress group based on Dunnett’s test at 3 and 6 h and Steel’s test at 24 h.

Intracerebroventricular (ICV) administration of the CRFR1 antagonists NBI27914 significantly ameliorated the decrease in food intake that occurred after novelty stress exposure in aged male mice at 3 and 6 h after stress exposure (p = 0.005 and p = 0.074, respectively). In contrast, the CRFR2 antagonist astressin 2B showed a negligible effect at 3 and 6 h after novelty stress exposure but tended to partially restore the decrease in food intake after 24 h (p = 0.051). ICV administration of the MC4R antagonist HS014 also significantly restored food intake at 3 and 6 h (p = 0.028 and p = 0.009, respectively) after novelty stress exposure (Fig. 2).

### Antagonistic effect of RKT components against CRFR1

Among the 36 RKT components assayed, glycycoumarin, nobiletin, tangeretin, isoliquiritigenin, [8]-shogaol, glycyrrhetic acid, and hesperetin exhibited inhibitory effects on the ovine CRF-induced increase in cAMP levels in CRFR1-transfected cells. The corresponding IC_50_ and *K*_B_ values are provided in Table 1. The same 36 RKT components showed low binding affinities for MC4R (data not shown).

### Determination of plasma concentrations of RKT components after a single oral administration of RKT in rats

**Figure 3:**
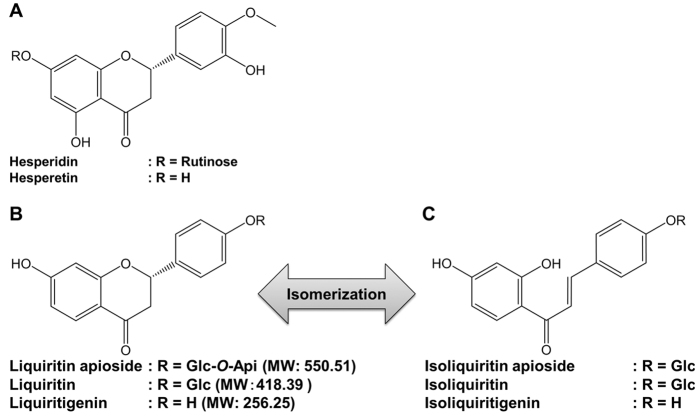
Chemical structures of rikkunshito components. (**A**) Hesperetin and its glycoside. (**B**) Liquiritigenin and its glycosides. (**C**) Isoliquiritigenin and its glycosides. Api, apiose; Glc, glucose.

The plasma levels of RKT components that demonstrated CRFR1 (Table 1) or 5-HT_2C_R antagonistic effects (Table 2)^13^ were investigated after a single oral administration of RKT at a dose of 1000 mg kg^−1^ in rats (Table 3). The ameliorative effect of RKT on hypophagia in young mice has been previously reported in several studies^18^,^23^. Therefore, we investigated the distribution of RKT components in young animals in the present study. We used rats to obtain a sufficient amount of plasma for our investigation. Because isoliquiritigenin and liquiritigenin have been reported to be chemically interchangeable^24^,^25^, we also examined the plasma levels of liquiritigenin and its glycoside forms that are present in RKT at high levels^22^. The chemical structures of hesperetin and its glycoside (hesperidin) are shown in Fig. 3A; those of liquiritigenin and its glycosides (liquiritin and liquiritin apioside) are shown in Fig. 3B; and those of isoliquiritigenin and its glycosides (isoliquiritin and isoliquiritin apioside) are shown in Fig. 3C. Among these components, the plasma concentration of liquiritin was highest with *C*_max_ being observed at 0.5 h after administration, followed by liquiritin apioside. Hesperetin, glycycoumarin and isoliquiritigenin were detected at low levels in plasma. Tangeretin, glycycoumarin and [8]-shogaol were below the detection limits.

### Brain distribution of hesperetin and isoliquiritigenin

**Figure 4:**
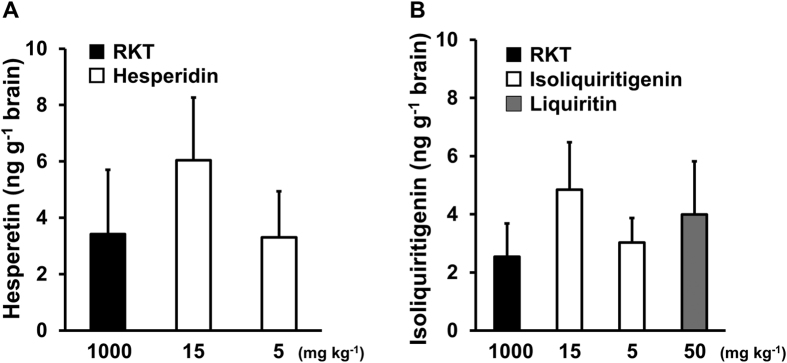
Brain levels of hesperetin and isoliquiritigenin after the oral administration of rikkunshito (RKT). (**A**) Hesperetin levels in the whole brain were determined in rats 3 h after a single oral administration of RKT (1000 mg kg^−1^, *n* = 4) and hesperidin (glycoside form of hesperetin, 5 and 15 mg kg^−1^, *n* = 4, 5). (**B**) Isoliquiritigenin levels in the whole brain were measured in rats 3 h after a single oral administration of RKT (1000 mg kg^−1^, *n* = 5), isoliquiritigenin (5 and 15 mg kg^−1^, *n* = 5) or liquiritin (50 mg kg^−1^, *n* = 3). The data are presented as the mean ± SEM.

Among the RKT components that were detected in plasma, we chose to determine the levels of hesperetin and isoliquiritigenin in the brain because they exhibited antagonistic effects on both CRFR1 and 5-HT_2C_R. Although the antagonistic activity of hesperetin was low, hesperidin showed the highest level in the RKT extract among the components of RKT^22^,^26^. We collected brain samples at 3 h after oral administration because RKT was most effective at 3 h, as shown in Fig. 1. Typical chromatograms of the authentic standards, blank, and brain sample are shown in Supplementary Figs S1 and S2. Hesperetin was detected in rat brains after the oral administration of RKT (1000 mg kg^−1^) or hesperidin (5 and 15 mg kg^−1^) (Fig. 4A). Isoliquiritigenin was also detected after the oral administration of RKT or isoliquiritigenin itself at 5 and 15 mg kg^−1^ (Fig. 4B). Furthermore, when we administered liquiritin at 50 mg kg^−1^, a comparable amount of isoliquiritigenin was detected in the brain (Fig. 4B).

## Discussion

In this study, we showed that 1) several components of RKT exhibited antagonistic activities against CRFR1; 2) hesperetin (Citri Unshiu Pericarpium-derived) and isoliquiritigenin (Glycyrrhizae Radix-derived), which are major active components of RKT that possess both CRFR1 and 5-HT_2C_R antagonistic activities, were distributed in the plasma and brain after a single oral administration of RKT; and 3) CRFR1 and MC4R antagonists, in addition to RKT and a 5-HT_2C_R antagonist, restored the initial decrease in food intake that occurred after novelty stress exposure in aged mice. Based on these results, we conclude that the ameliorative effect of RKT in this model is at least partly attributable to brain-distributed active components possessing CRFR1 and 5-HT_2C_R antagonistic activities.

We have previously shown that RKT ameliorates the occurrence of prolonged hypophagia after novelty stress exposure in aged mice^13^,^14^. In the present study, we investigated the effect of RKT at 1 and 3 h after novelty stress exposure because we considered it important to investigate the effects in early stages to clarify the involvement of various neurotransmitters in the initiation of hypophagia after stress exposure in aged mice. A marked decrease in food intake was restored by the oral administration of RKT and a 5-HT_2C_R antagonist 1 h after novelty stress exposure in this study. This indicates that RKT and 5-HT_2C_R antagonists exert ameliorative effects, not only in the prolonged stage, but also in the early stage after novelty stress exposure in aged mice.

CRF and 5-HT play significant roles in the stress response and the regulation of feeding behaviors. Multiple lines of evidence indicate that there is a positive correlation between 5-HT_2C_R and CRF neuron activation. CRF-containing neurons in the PVN have been reported to be consistently depolarized in the presence of a high-affinity 5-HT_2C_R agonist^17^ and CRF neurons activation in response to an antigenic stimulus was blunted in 5-HT_2C_R knockout mice^27^. CRF neuron activation during stress causes suppression of feeding behavior^28^,^29^. We investigated the effect of CRFR antagonists on novelty stress-induced hypophagia in aged male mice and found that ICV administration of CRFR1 antagonists, but not CRFR2 antagonists, inhibited the initial decrease in food intake. These results indicate that the activation of 5-HT_2C_R-CRF neuron-CRFR1 is involved in the initiation of hypophagia after novelty stress exposure in aged mice. Additionally, the activation of 5-HT_2C_R-CRF neuron-CRFR2 may be partly involved in sustained hypophagia, as the CRFR2 antagonist demonstrated a partial ameliorative effect only at 24 h after stress exposure.

**Figure 5:**
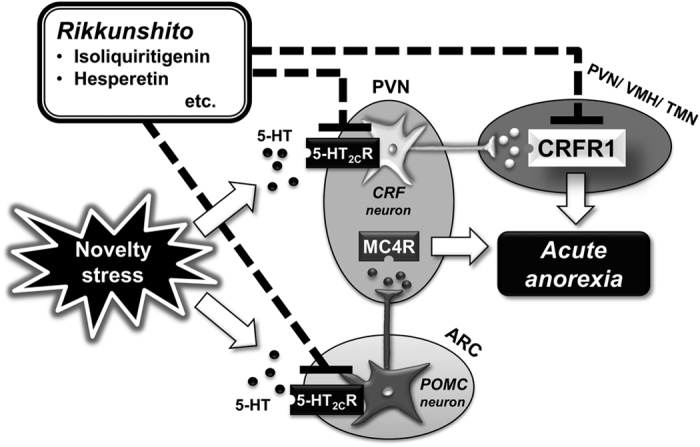
A hypothesis to explain the ameliorative effect of rikkunshito on novelty stress-induced anorexia. Activation of 5-HT_2C_R induces CRF neuron and POMC neuron activation and causes appetite loss after novelty stress exposure in aged male mice. RKT is assumed to exert its ameliorative effect via 5-HT_2C_R antagonism and CRFR1 antagonism in the brain. CRF, corticotropin-releasing factor; CRFR1, CRF type-1 receptor; 5-HT, serotonin; 5-HT_2C_R, 5-HT_2C_ receptor; MC4R, melanocortin-4 receptor; POMC, pro-opiomelanocortin; PVN, paraventricular nucleus; ARC, arcuate nucleus; VMH, ventromedial hypothalamic nucleus; TMN, tuberomammillary nucleus.

In the present study, we showed that ICV administration of an MC4R antagonist also inhibited the initial decrease in food intake induced by novelty stress exposure in aged mice. These results suggest that CRFR1 and MC4R activation resulting from anorexigenic CRF and POMC neuron activation is involved in the initiation of hypophagia induced by novelty stress exposure in aged mice (Fig. 5).

In the present study, we found that seven components of RKT showed antagonistic effects on CRFR1 (Table 1). To the best of our knowledge, this study is the first to report the antagonistic activities of these components against CRFR1. The same 36 RKT components showed low binding affinities for MC4R. Therefore, we assumed that the ameliorative effect of RKT was attributable to CRFR1 antagonism in addition to the 5-HT_2C_R antagonistic effect (Fig. 5).

In order to test our hypothesis that the antagonism of central CRFR1 and 5-HT_2C_R is involved in the ameliorative effect of RKT, we first confirmed that the RKT components possessing either CRFR1 or 5-HT_2C_R antagonistic activity were distributed in plasma. We also confirmed the brain distribution of hesperetin and isoliquiritigenin, which are components that possess both CRFR1 and 5-HT_2C_R antagonistic activities, after oral administration of RKT. Isoliquiritigenin has been reported to show an ameliorative effect on hypophagia after novelty stress in young mice^18^. Additionally, both isoliquiritigenin and hesperidin (the glycoside form of hesperetin) have also been observed to improve cisplatin-induced hypophagia in rats via 5-HT_2C_R antagonism^15^,^30^. Therefore, the brain distribution of these compounds after oral administration of RKT suggests that these components may exert their antagonistic effects on CRFR1 and 5-HT_2C_R in the brain and that they partially contribute to the ameliorative effect of RKT on hypophagia after novelty stress exposure.

In the present study, we detected isoliquiritigenin in brain tissue after the oral administration of RKT (1000 mg kg^−1^), and the levels in the brain were comparable to those observed after the oral administration of isoliquiritigenin itself at a dose of 5 mg kg^−1^. This level was much higher than expected, as isoliquiritigenin has been estimated to occur at approximately 0.019 mg per 1000 mg of RKT water extract^22^. Two types of its glycoside forms (isoliquiritin and isoliquiritin apioside) have been reported to occur at approximately 10 times the level of isoliquiritigenin itself in RKT^26^,^22^. Moreover, liquiritin and liquiritin apioside (glycoside form of liquiritigenin) have been reported to occur at 70–80 times the level of isoliquiritigenin in RKT^22^. As we detected isoliquiritigenin in the brain after liquiritin administration, a portion of the liquiritin and liquiritin apioside present in RKT may have become hydrolyzed and isomerized and been distributed in the brain as isoliquiritigenin.

The levels of hesperetin and isoliquiritigenin in the brain after oral administration of RKT may be insufficient relative to their *K*_B_ values to exert any pharmacological effect against CRFR1 and 5-HT_2C_R (almost one magnitude lower in the case of isoliquiritigenin). This implies that hesperetin and isoliquiritigenin alone are not sufficient to fully account for the effects of RKT. In the present study, we only measured the brain levels of two representative active components that possess both CRFR1 and 5-HT_2C_R antagonistic activities. Additionally, we showed that there are several other components that possess CRFR1 antagonistic effects *in vitro*, suggesting that these components might also exert antagonistic activities *in vivo*, although we did not investigate the brain distribution in this study. One such component is glycycoumarin, which has been reported to ameliorate hypophagia after novelty stress exposure in young mice^18^. We also confirmed the *in vivo* effect of glycyrrhizin, the glycoside form of glycyrrhetic acid. Glycyrrhetic acid was shown to possess the highest *in vitro* CRFR1 antagonistic activity among the components evaluated in the present study and to exhibit no antagonistic activity against 5-HT_2C_R. Glycyrrhizin administration significantly restored the decreased food intake observed after novelty stress exposure in young mice (Supplementary Fig. S3). The extensive distribution of glycyrrhetic acid in the brain after an oral administration of yokukansan extract containing Glycyrrhizae Radix^31^ suggests that glycyrrhetic acid is distributed at high levels after RKT administration. Therefore, these additional components possessing CRFR1 antagonistic activity may also have contributed to the ameliorative effect of RKT on hypophagia. Because CRFR1 antagonistic effects were detected in the present study, it will be necessary to investigate of contribution of nobiletin and tangeretin to the effects of RKT and their specific brain distribution in further studies, as they have been reported to be distributed in the brain *in vivo*^32^,^33^,^34^. Moreover, we only investigated 36 components of RKT in this study, and there may be other components that possess greater antagonistic activities against 5-HT_2C_R, CRFR1 or MC4R and that are distributed in the brain at higher levels. A non-targeted analysis to conduct a search for other active components will be necessary in further studies. In addition, there is a possibility that a combination of various active components that show multiple mechanisms of action may exert enhanced effects relative to the effect of a single component with a single target.

In the present study, we demonstrated the ameliorative effect of RKT in aged mice. Because the BBB in aged animals is reported to be more permeable than in younger animals^35^,^36^, greater amounts of active components may be distributed in the brains of aged animals than in younger animals. Further precise pharmacokinetic studies of RKT components in aged animals are required to examine those differences.

In conclusion, we demonstrated that the initiation of novelty stress-induced anorexia in aged mice is due to 5-HT_2C_R and CRFR1/MC4R activation. Our results also indicate that brain distributed components of RKT possessing CRFR1 and 5-HT_2C_R antagonistic activities may be partly involved in its ameliorative effect in this model. However, further investigation of other active components or combinations of these components is required to fully understand the effects of RKT.

## Materials and Methods

### Animals

Male C57BL/6 mice at 77 ± 8 weeks of age and 6- to 7-week-old male Sprague Dawley rats were purchased from Charles River Laboratories Japan (Tokyo, Japan). We used mice before the onset of ageing cachexia. Before the experiments, five mice per cage or four to five rats per cage were acclimated in a temperature and humidity-controlled room under a 12-h light–dark cycle with free access to food and water. This study was approved by and conducted according to the guidelines of the experimental animal ethics committees of Tsumura & Co. (Ibaraki, Japan, approved protocol No.: 11–134, 11–135, 12–113, 12–135).

### Rikkunshito

RKT (Tsumura & Co.) was obtained by spray drying a hot water extract of a mixture of eight crude drugs: Atractylodis lanceae rhizoma (4.0 g), Ginseng radix (4.0 g), Pinelliae tuber (4.0 g), Poria (4.0 g), Zizyphi fructus (2.0 g), Citri Unshiu Pericarpium (2.0 g), Glycyrrhizae radix (1.0 g) and Zingiberis rhizoma (0.5 g). The major active components in 1 g of ‘Tsumura rikkunshito extract granules for prescription’ (1 g contains 0.53 g of the spray-dried hot water extract and pharmaceutical excipients) from a specific lot have been reported to be hesperidin, 3750 μg; glycyrrhizin, 1370 μg; narirutin, 932 μg; liquiritin, 801 μg; liquiritin apioside, 697 μg; isoliquiritin, 101 μg; isoliquiritin apioside, 85.2 μg; liquiritigenin, 79.8 μg; pachymic acid, 67.5 μg; atractylodin, 56.3 μg; heptamethoxyflavone, 23.4 μg; nobiletin, 17.4 μg; isoliquiritigenin, 9.85 μg; naringenin, 3.95 μg and glycyrrhetic acid, 1.84 μg in a certain lot^22^.

### Exposure to novelty stress

The novelty stress-induced hypophagia test can be used to evaluate the degree of anxiety or depression by evaluating the suppression of food intake after exposure to a novel environment^37^. Testing was performed as previously described^13^. Group-housed mice (five mice per cage) were suddenly transferred to separate cages (one mouse per cage) after overnight fasting. Control mice were housed in separate cages for 7 days before the beginning of the experiments. Provisional food intake by the group-housed mice was calculated to ensure that no differences were observed between the group-housed and control mice^13^.

### Effects of tested drugs on anorexia induced by novelty stress exposure in aged male mice

The selective 5-HT_2C_R antagonist, SB242084 (6 mg kg^−1 ^38,39; Sigma–Aldrich, St. Louis, MO, USA) and RKT (1000 mg kg^−1^) suspended in distilled water were orally administered to overnight fasted mice immediately after they were isolated (*n* = 5). The administered dose of RKT is approximately equivalent to a typical clinical dose when converted based on body surface area (as recommended by the FDA) and this dose has previously been reported to show ameliorative effects in a mouse model of anorexia^13^,^14^,^40^,^41^. Cumulative food intake was determined at 1, 3 and 6 h after stress exposure. We previously confirmed that oral administration of SB242084 (6 mg kg^−1^) and RKT (1000 mg kg^−1^) to unstressed-young or aged mice does not affect food intake^13^.

To investigate the involvement of CRFR and MC4R on decreased food intake, the CRFR1 antagonist, NBI27914 (Sigma–Aldrich, 10 μg mouse^−1 ^18,42), the CRFR2 antagonist astressin 2B (Sigma–Aldrich, 10 μg mouse^−1 ^43,18) and the MC4R antagonist HS014 (Sigma–Aldrich, 0.15 μg mouse^−1 ^44) were dissolved in physiological saline and administered via ICV injection immediately after novelty stress exposure, and food intake was subsequently measured. ICV administration was performed according to the method reported by Haley and McCormick^45^, with a slight modification. A 26-gauge stainless-steel needle attached to PE-10 tubing fitted to a 10-μL microsyringe was inserted into the brain (2.6 mm below the surface of the skull, 1.0 mm lateral and 0.5 mm anterior to the bregma) of lightly held mice. The injections were performed without anesthesia to avoid any short-time effects of anesthesia on food intake. An injection volume of 5 μL was administered over 30 s. Mice that showed visible bleeding or abnormal behaviors following ICV administration were excluded from the analysis because of the inadequacy of the administration procedure. Direct ICV administration was performed by a skilled individual who have confirmed the location of ICV injection through dye infusions with a success rate of 97%.

### *In vitro* functional assay of CRFR1 antagonistic activity of RKT components

Evaluation of the antagonistic activity of various compounds against human CRFR1 in transfected CHO cells was performed at Cerep (Paris, France) by measuring the effects of the compounds on agonist-induced cAMP production via the HTRF detection method, as previously reported^46^. Briefly, the cells were pre-incubated in the presence of the test compound for 5 min at room temperature, and the reference agonist ovine CRF was added (10 nmol L^−1^), followed by 30 min of incubation at 37 °C. The cells were then lysed, and the fluorescence acceptor (D2-labelled cAMP) and fluorescence donor (anti-cAMP antibody labelled with europium cryptate) were added. After 60 min at room temperature, fluorescence transfer was measured (*λ*_ex_ = 337 nm and *λ*_em_ = 620 and 665 nm), and the cAMP concentration was determined by dividing the signal measured at 665 nm by that measured at 620 nm (ratio). The results are expressed as the percent inhibition of the control response to the reference agonist. A concentration-response curve was generated to calculate IC_50_ values. The apparent dissociation constants (*K*_B_) were calculated using the modified Cheng-Prusoff equation (*K*_B_ = IC_50_/(1 + (*A*/EC50A)), where *A* = the concentration of the reference agonist in the assay and EC50A = the EC_50_ value of the reference agonist).

### Binding assay of RKT components with MC4R receptors

Evaluation of the binding activity at human MC4R in transfected HEK-293 cells was performed at Ricerca Biosciences, LLC (Taipei, Taiwan) as previously reported^47^,^48^. Briefly, transfected cells were incubated for 2 h at 37 °C with 0.05 ml of binding buffer in each well, which also contained 0.02 nM [^125^I] NDP-α-MSH and a 100 μM concentration of the test compounds. All assays were performed in duplicate. After incubation, the cells were washed and detached from the plates, and radioactivity was counted.

### Determination of plasma levels of active components after the oral administration of RKT in rats

Rats fasted overnight were orally administered 1000 mg kg^−1^ of RKT and decapitated at 30, 60 and 120 min to collect blood samples (*n* = 3). The blood was also collected from untreated rats as a control (*n* = 3). Blood samples were heparinized and centrifuged at 3000 rpm for 10 min. Plasma samples were collected and stored at −80 °C until use. Determination of the plasma concentrations of the active components was performed by Sumika Chemical Analysis Service Ltd. (Osaka, Japan), as previously reported^22^. The method is summarized in the Supplementary Method.

### Brain distributions of hesperetin and isoliquiritigenin after the oral administration of RKT or its components in rats

Rats were orally administered RKT (1000 mg kg^−1^), hesperidin (5, 15 mg kg^−1^), isoliquiritigenin (5, 15 mg kg^−1^) and liquiritin (50 mg kg^−1^) and decapitated 3 h after administration. The brain (the cerebrum except for the cerebellum, medulla oblongata and olfactory bulb) was the collected and immediately frozen and stored at −20 °C until use. The brain was homogenized after adding 3 volumes (w/w) of distilled water.

For the preparation of calibration standards, each standard substance and 3-hydroxy chalcone (used as the internal standard [IS]) were separately dissolved in methanol to prepare standard stock solutions (100 μg ml^−1^). After mixing 0.1 ml of each standard stock solution except for the IS solution, the mixed solution was diluted with 50% acetonitrile to a final volume of 10 ml, which was the standard stock solution A (1 μg ml^−1^). Standard working solutions at concentrations ranging from 1 to 100 ng ml^−1^ were prepared by further diluting standard stock solution A and adding IS solution (0.1 ml). The concentrations of the standard working solutions used for calibration curve matching of the matrix were 1, 5, 10, 50 and 100 ng ml^−1^. All of the stock solutions and working solutions were stored at −20 °C.

For isoliquiritigenin measurements, brain samples and calibration standards were extracted using ice cold MeOH/MeCN (1/1, v/v). Next, 400 μl of acetonitrile and 10 μl of IS solution were added to the samples and calibration standards. The samples were subsequently mixed for 3 min and centrifuged at 10,000 rpm for 5 min. The supernatant was transferred to a clear test tube and evaporated to dryness under a gradual nitrogen flow at 37 °C. The dried residue was reconstituted in 200 μl of 0.1% TFA containing 20% MeCN. A 50-μl aliquot of the solution was then injected into the HPLC system for analysis. For measurements of hesperetin, 10 μl of IS solution was added to the samples and calibration standards. The brain samples and calibration standards were then deproteinized with ice-cold trichloroacetic acid, followed by mixing for 3 min and centrifugation at 10000 rpm for 5 min. Finally, 50 μl of the supernatant was injected into the HPLC system for analysis.

For liquid chromatography, an LA-10ADvp system (Shimadzu) was used for solvent and sample delivery. A Symmetry C18 column (100 × 4.6 mm I.D., 3-μm particle size; Waters, Milford, MA) was employed to separate each analyte at 40 °C. The mobile phase consisted of solution A (0.1% TFA, v/v) and solution B (acetonitrile), and a gradient of solution B (10%, 0–5 min; 70%, 20 min; 70%, 25 min; and 10%, 25.1–30 min; v/v) at a flow rate of 0.9 ml/min was used during the analysis. The detection wavelengths were UV 285 nm for hesperetin and 369 nm for isoliquiritigenin.

### Statistical analyses

Statistical analyses of mean values were performed with Dunnett’s test or Steel’s test vs. the stress group after applying Bartlett’s test using StatLight 2000(C) (Yukms Co. Ltd.). The data are presented as the mean ± standard error for each group, and *p* < 0.05 was considered to indicate statistical significance.

## Additional Information

**How to cite this article**: Mogami, S. *et al.* CRF receptor 1 antagonism and brain distribution of active components contribute to the ameliorative effect of rikkunshito on stress-induced anorexia. *Sci. Rep.* **6**, 27516; doi: 10.1038/srep27516 (2016).

## Supplementary Material

Supplementary Information

## Figures and Tables

**Table 1:** Antagonistic effects of rikkunshito components against CRFR1.

Components	Crude drug	IC_50_ (μmol/L)	*K*_B_ (μmol/L)
Nobiletin	Citri Unshiu Pericarpium	15	0.36
Tangeretin	Citri Unshiu Pericarpium	60	1.5
Hesperetin	Citri Unshiu Pericarpium	130	45
Isoliquiritigenin	Glycyrrhizae Radix	28	0.67
Glycycoumarin	Glycyrrhizae Radix	52	1.3
Glycyrrhetic acid	Glycyrrhizae Radix	14	0.35
[8]-Shogaol	Zingiberis Rhizoma	74	1.8

**Table 2:** Antagonistic effects of rikkunshito components against 5-HT_2C_R.

Components	Crude drug	IC_50_ (μmol/L)
Hesperetin	Citri Unshiu Pericarpium	48.2*
Isoliquiritigenin	Glycyrrhizae Radix	5.5*
Glycycoumarin	Glycyrrhizae Radix	7.7*
[8]-Shogaol	Zingiberis Rhizoma	36.9*

^*^IC_50_ (μmol/L) values for the cell function assay have been previously reported by Nahata *et al.*^13^.

**Table 3:** Plasma concentrations after the oral administration of rikkunshito at 1000 mg kg^−1^ in rats.

Compound	Crude drug	Plasma concentration (ng/ mL)		
		0.5 h	1 h	2 h
Nobiletin	Citri Unshiu Pericarpium	0.492 ± 0.190	0.127 ± 0.0306	0.0225 ± 0.000285
Tangeretin	Citri Unshiu Pericarpium	<0.020	<0.020	<0.020
Hesperetin	Citri Unshiu Pericarpium	0.192 ± 0.192	<0.100	<0.100
liquiritin apioside	Glycyrrhizae Radix	2.60 ± 0.699	1.73 ± 0.0985	0.913 ± 0.359
liquiritin	Glycyrrhizae Radix	2.84 ± 0.968	1.11 ± 0.0748	0.309 ± 0.213
Isoliquiritigenin	Glycyrrhizae Radix	0.392 ± 0.000667	0.267 ± 0.0361	0.174 ± 0.0347
Glycyrrhetic acid	Glycyrrhizae Radix	2.00 ± 1.68	0.396 ± 0.202	17.7 ± 16.6
Glycycoumarin	Glycyrrhizae Radix	<0.020	<0.020	<0.020
[8]-Shogaol	Zingiberis Rhizoma	<0.100	<0.100	<0.100
