# Supplementary material for: CRF receptor 1 antagonism and brain distribution of active components contribute to the ameliorative effect of rikkunshito on stress-induced anorexia
Source: Sci Rep. 2016 Jun 8;6:27516. doi: 10.1038/srep27516 (PMC4897628; doi:10.1038/srep27516)
Supplement: Supplementary Information [file srep27516-s1.pdf]

Title

CRF receptor 1 antagonism and brain distribution of active components contribute to the ameliorative effect of rikkunshito on stress-induced anorexia

Authors

Sachiko Mogami [a], Chiharu Sadakane [a], Miwa Nahata [a], Yasuharu Mizuhara [a], Chihiro Yamada [a], Tomohisa Hattori [*a], Hiroshi Takeda [b,c]

a Tsumura & Co., Tsumura Research Laboratories, Ibaraki 300-1192, Japan

b Pathophysiology and Therapeutics, Hokkaido University Faculty of Pharmaceutical Sciences, Sapporo, Hokkaido 060-0812, Japan

c Gastroenterology and Hepatology, Hokkaido University Graduate School of Medicine, Sapporo, Hokkaido 060-8638, Japan

*Address for Correspondence:

Tomohisa Hattori

Tsumura Research Laboratories, Tsumura & Co., 3586 Yoshiwara, Ami-machi, Inashiki-gun, Ibaraki 300-1192, Japan.

Tel.: +81 29 889 3855;

Fax: +81 29 889 2158.

E-mail address: hattori_tomohisa@mail.tsumura.co.jp

Supplementary Figure S1.

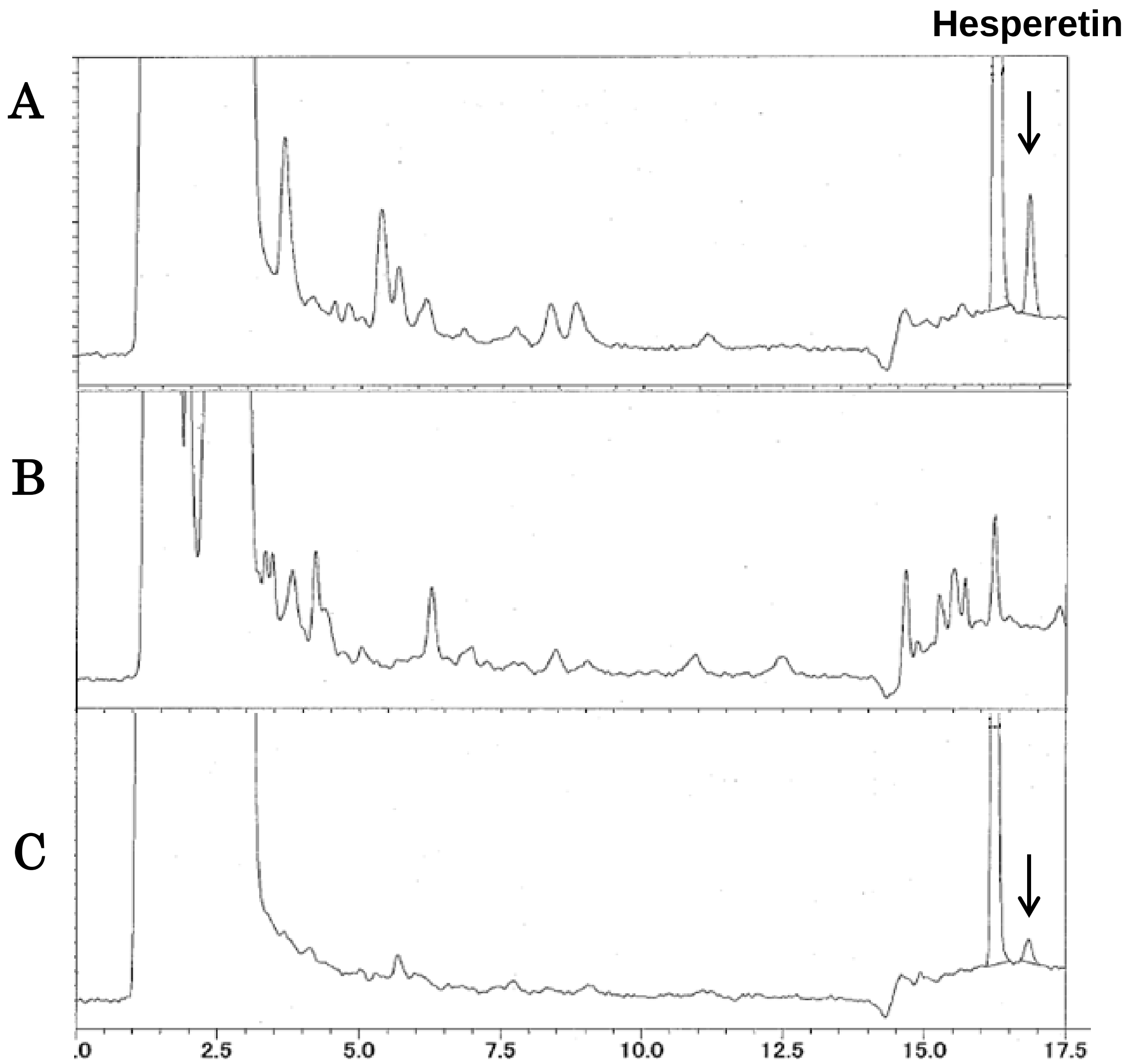

HPLC chromatograms of authentic standards, blank, and brain sample for hesperetin. (A) Brain sample spiked with standard solution. (B) Brain sample obtained from rats not treated with rikkunshito. (C) Brain sample after oral administration of rikkunshito.

Supplementary Figure S2.

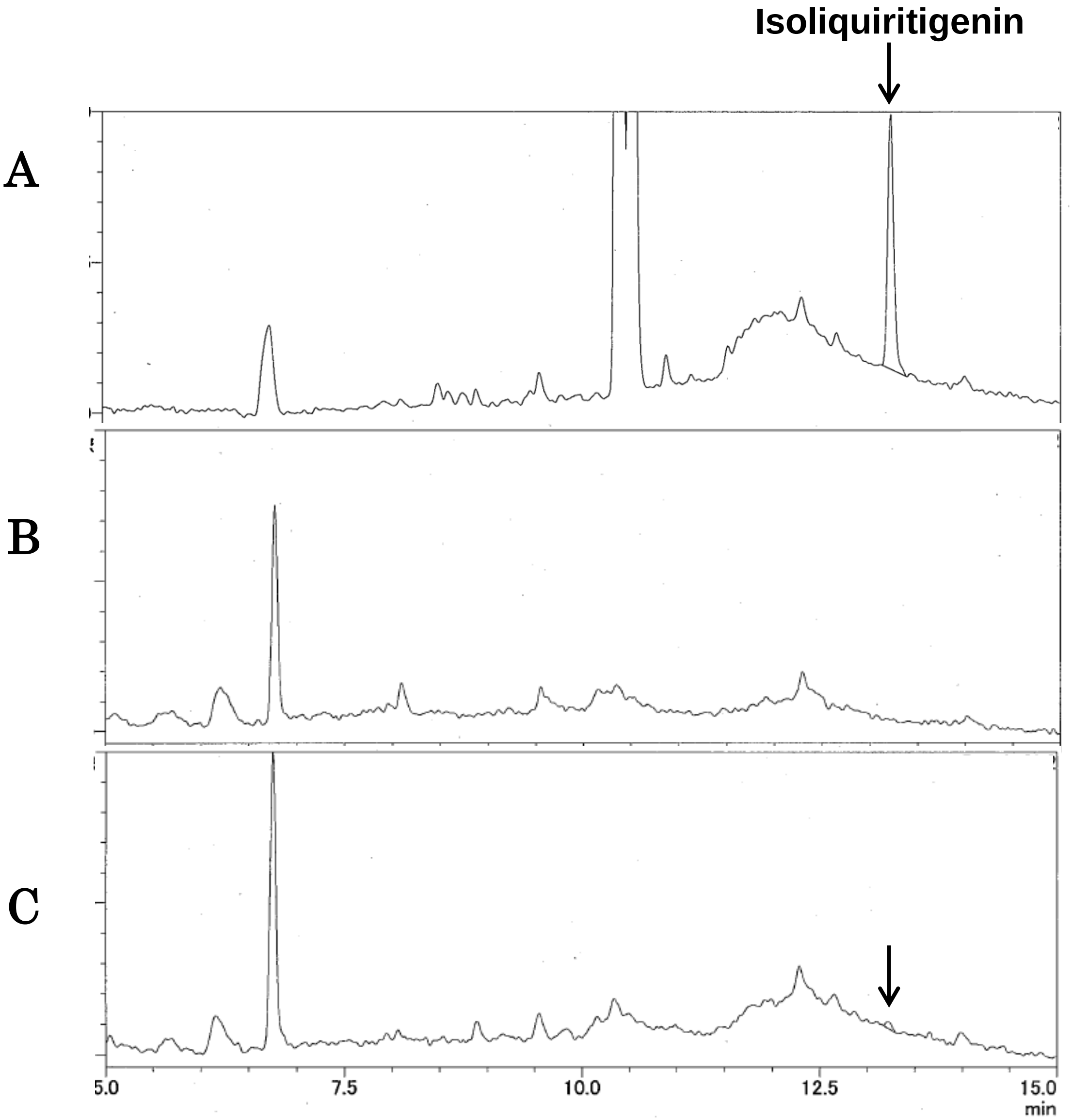

HPLC chromatograms of authentic standards, blank, and brain sample for isoliquiritigenin. (A) Brain sample spiked with standard solution. (B) Brain sample obtained from rats not treated with rikkunshito. (C) Brain sample after oral administration of rikkunshito.

Supplementary Figure S3.

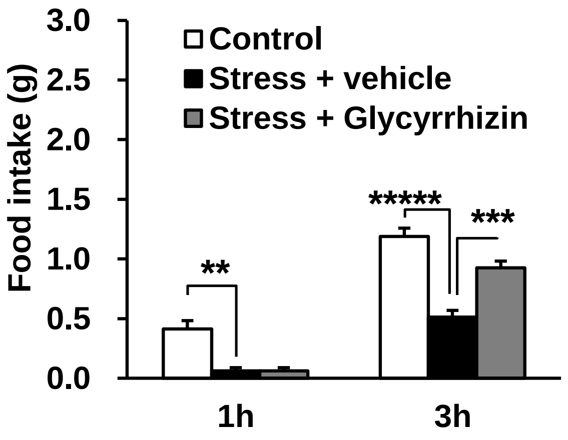

Effect of glycyrrhizin (glycoside form of glycirrhetic acid) on novelty stress-induced hypophagia. Six week-old male mice were deprived of food for 24 h and orally administered with glycyrrhizin (4mg/ kg). Immediately after administration, the mice were isolated and cumulative food intake was determined at 1 and 3 h after the stress exposure. Data are presented as the mean ± SEM (n = 8). **, ***,***** $p < 0.01$, 0.001, 0.00001 vs. stress group by Steel test at 1 h and Dunnett test at 3 h.

Supplementary Method

Determination of plasma levels of active components after the oral administration of RKT in rats

Twenty five microliters of methanol was added to 500 μL plasma samples followed by mixing. Atractylenolide III or digoxin (internal standard [IS]) solution (50 μL) was added to the solutions followed by mixing. Ammonium acetate solution (100 mmol L^{-1}, 100 or 400 μL) was added to the solutions followed by mixing. A solid-phase cartridge (OASIS HLB, 30 mg/1 cc; Waters, Milford, MA, USA) was conditioned with methanol and 10 mmol L−1 ammonium acetate solution, and the samples were loaded onto the cartridge. The cartridge was washed with water: methanol (9:1, v/v), and 1.5 mL of methanol and 30 μL of propylene glycol were added to the cartridge to elute the analytes. The mixtures were dried at 25°C under a stream of nitrogen gas. Furthermore, 100 μL of 10 mmol L−1 ammonium acetate: methanol (8:2, v/v) was added to the dried samples followed by mixing and sonication. The solutions were filtered (0.22 μm) and followed by injection into an LC–MS/MS system. This system comprised an LC-20A system (Shimadzu, Kyoto, Japan) connected to an API5000 triple quadrupole mass spectrometer fitted with a TurboIonSpray electrospray ionization interface (AB Sciex, Framingam, MA, USA). Those analytical conditions are shown in Supplementary Tables S1 and S2. The standard components contained in RKT were supplied by Tsumura & Co.

Table S1 Methods of LC-MS/MS: Ion parameters of rikkunshito components and internal standards

Compound name	Q1Mass (*m/z*)	Q3Mass (*m/z*)	Polarity	LC methods ID
**Glycyrrhetic acid**	471	149	Positive	1
**Nobiletin**	403	373	Positive	1
**Tangeretin**	373	343	Positive	1
**[8]-Shogaol**	303	167	Negative	2
**Liquiritin apioside**	550	255	Negative	2
**Liquiritin**	417	255	Negative	2
**Isoliquiritigenin**	255	119	Negative	2
**Glycycoumarin**	367	309	Negative	2
**Hesperetin**	301	164	Negative	2
**Digoxin (internal standard)**	780	651	Negative	2

Table S2 Methods of LC-MS/MS: HPLC conditions for analyzing RKT components

Methods ID		HPLC condition
1	Column:	shim-pack XR-ODS II (2.0 mm I.D., × 100 mm L., 2.2-μm particle size; Shimadzu GLC Ltd., Tokyo, Japan)
	Mobile phase	(A) 10 mM ammonium acetate, (B) methanol
	Gradient elution program (%B in A)	0.01–0.50 min, 20%; 0.50–2.50 min, 20–40%; 2.50–17.00 min, 40–85%; 17.00–30.00 min, 85–95%; 30.00–34.00 min, 95%; 34.10–40.00 min, 20%
	flow rate	0.2 mL/min
	column temperature	40°C
	injection volume	10 μL
2	Column:	shim-pack XR-ODS II (2.0 mm I.D., × 100 mm L., 2.2-μm particle size; Shimadzu GLC Ltd., Tokyo, Japan)
	Mobile phase	(A) 10 mM ammonium acetate, (B) methanol
	Gradient elution program (%B in A)	0.01–0.50 min, 20%; 0.50–2.50 min, 20–40%; 2.50–17.00 min, 40–85%; 17.00–30.00 min, 85–95%; 30.00–34.00 min, 95%; 34.10–40.00 min, 20%
	flow rate	0.2 mL/min
	column temperature	40°C
	injection volume	30 μL
